# Making head and neck cancer clinical data Findable-Accessible-Interoperable-Reusable to support multi-institutional collaboration and federated learning

**DOI:** 10.1093/bjrai/ubae005

**Published:** 2024-03-06

**Authors:** Varsha Gouthamchand, Ananya Choudhury, Frank J P Hoebers, Frederik W R Wesseling, Mattea Welch, Sejin Kim, Joanna Kazmierska, Andre Dekker, Benjamin Haibe-Kains, Johan van Soest, Leonard Wee

**Affiliations:** Clinical Data Science, Faculty of Health Medicine and Life Sciences, Maastricht University, Maastricht 6229 EN, The Netherlands; Clinical Data Science, Faculty of Health Medicine and Life Sciences, Maastricht University, Maastricht 6229 EN, The Netherlands; Dept of Radiation Oncology (MAASTRO), School of Oncology and Reproduction, Maastricht University Medical Centre+, Maastricht 6229 ET, The Netherlands; Dept of Radiation Oncology (MAASTRO), School of Oncology and Reproduction, Maastricht University Medical Centre+, Maastricht 6229 ET, The Netherlands; Radiation Medicine Program, Princess Margaret Cancer Centre, University Health Network, Toronto, ON M5G 2C4, Canada; Cancer Digital Intelligence, Princess Margaret Cancer Centre, Toronto, ON M5G 2C4, Canada; Dept of Radiation Oncology, Greater Poland Cancer Centre II, Poznan 61-866, Poland; Clinical Data Science, Faculty of Health Medicine and Life Sciences, Maastricht University, Maastricht 6229 EN, The Netherlands; Dept of Radiation Oncology (MAASTRO), School of Oncology and Reproduction, Maastricht University Medical Centre+, Maastricht 6229 ET, The Netherlands; Medical Biophysics University of Toronto, Vector Institute for Artificial Intelligence and Ontario Institute for Cancer Research, Toronto, ON M5G 0C6, Canada; Brightlands Institute for Smart Society, Faculty of Science and Engineering, Maastricht University, Heerlen 6411 CR, The Netherlands; Clinical Data Science, Faculty of Health Medicine and Life Sciences, Maastricht University, Maastricht 6229 EN, The Netherlands

**Keywords:** FAIR data, Semantic Web, ontologies, RDF, federated learning, artificial intelligence

## Abstract

**Objectives:**

Federated learning (FL) is a group of methodologies where statistical modelling can be performed without exchanging identifiable patient data between cooperating institutions. To realize its potential for AI development on clinical data, a number of bottlenecks need to be addressed. One of these is making data Findable-Accessible-Interoperable-Reusable (FAIR). The primary aim of this work is to show that tools making data FAIR allow consortia to collaborate on privacy-aware data exploration, data visualization, and training of models on each other’s original data.

**Methods:**

We propose a “Schema-on-Read” FAIR-ification method that adapts for different (re)analyses without needing to change the underlying original data. The procedure involves (1) decoupling the contents of the data from its schema and database structure, (2) annotation with semantic ontologies as a metadata layer, and (3) readout using semantic queries. Open-source tools are given as Docker containers to help local investigators prepare their data on-premises.

**Results:**

We created a federated privacy-preserving visualization dashboard for case mix exploration of 5 distributed datasets with no common schema at the point of origin. We demonstrated robust and flexible prognostication model development and validation, linking together different data sources—clinical risk factors and radiomics.

**Conclusions:**

Our procedure leads to successful (re)use of data in FL-based consortia without the need to impose a common schema at every point of origin of data.

**Advances in knowledge:**

This work supports the adoption of FL within the healthcare AI community by sharing means to make data more FAIR.

## Introduction

Federated learning (FL) has been used to address data sharing for statistical modelling on cancer data^1–8^ without exchanging patient data between cooperating institutions. The Personal Health Train (PHT)^9^ is a manifesto for implementing FL that describes technological, governance and procedural components needed to execute a fully working FL infrastructure.

From a procedural point of view, PHT will not require an exchange of identifiable individual patient-level data between institutions to generate models or statistical insights. Governance is important in terms of compliance with local and international privacy laws, such as the General Data Protection Regulation,^10^ and the allocation of intellectual property rights between members of a collaboration. The legal agreement templates required to facilitate PHT collaborations have been made openly accessible.^11^ Technologically, PHT requires 3 interdependent components to be set up—“tracks” are protected telecommunications links needed to transmit messages between a central model aggregation hub and the participating institutions, “trains” are containerized software applications that carry a data query filter with some analysis code to be executed locally inside the institutional firewall, and “stations” are the institutional data repositories which holds the actual patient data being used to fit a single global multiinstitutional model.

To date, the major direction of FL research has been on the tracks and trains. This overshadows the fact that data preparation and data exploration are prerequisite steps before any statistical model or artificial intelligence application should be developed. The PHT especially calls for the data in the stations to first be made “FAIR”—Findable, Accessible, Interoperable, and Reusable—prior to performing any modelling or analysis.^12^ The FAIR principles define those attributes needed to make data usable by machine algorithms and by human operators.^13^ Without fixating on any kind of “master” schema or terminology, FAIR data gives a community of users, for example, researchers and cancer clinicians, a high degree of autonomy and interoperability without limiting reusability of each other’s data when for other studies. The FAIR data query must be executable with a single common query, even when each station uses a different language, different data schema and different clinical terminologies. The recent Web3.0 standards (Semantic Web)^14^ such as data graphs and data linking directly support the incorporation of the FAIR attributes into data.

The primary aim of this work is to show that public open-access software tools that make data FAIR allow PHT consortia to conduct privacy-aware data exploration, data visualization, and training of models. We show results consisting of a federated dashboard for data exploration and summarizing case-mix variables from multiple dispersed datasets, which allows clinicians and researchers to select consistent cohorts to study from each other’s datasets. We then show how to train a range of prognostic models using various formats of data (clinical factors and radiomic features). We selected a clinically meaningful use case in head and neck cancer outcomes prognostication, using a mixture of public open data and private institutional data, to make clear the clinical relevance of our work in realistic situations.

## Methods

Four public datasets were used; HN1,^15^ HEAD-NECK,^16^ OPC,^17^ and HNSCC^18^ obtained from The Cancer Imaging Archive.^19^ One private institutional dataset named HN3 was included with internal review board authorization from MAASTRO Clinic in The Netherlands. HN3 consisted of demographically the same population as HN1. Along with case-mix factors and long-term clinical follow-up, we retrieved radiotherapy data consisting of planning CT (in DICOM format) and primary gross tumour volume (GTV) delineations made by expert radiation oncologists (as DICOM-RTSTRUCT) for each of the above datasets. All GTV delineations were taken as approved for radiotherapy; there was no editing after retrieval. We distributed each of the HN1, HEAD-NECK, and HNSCC datasets into 3 virtual machines (VMs) in the Amazon Web Services cloud computing server based in Europe, that is, 1 dataset per VM. The datasets OPC and HN3 were locally hosted by institutions in Canada and The Netherlands, respectively.

### Data conversion and making data FAIR

Before any type of federated work, the each dataset had to be prepared locally. The clinical data was converted into Resource Descriptor Format (RDF),^20^ which is the primary building block of the Semantic Web Query and Retrieval Language (SPARQL),^21^ using an open-source software library known as *triplifier.*^22^ To minimize the amount of coding and scripting needed from a user, we provided a graphical user interface (GUI) that guides the local investigator (eg, a clinician) through the process one step at a time—first selecting either a CSV file or SQL relational database to import, and then *triplifier* automatically converts the contents of the imported database into an RDF file. At the same time, *triplifier* extracts the organizational schema of the imported database as a Web Ontology Language (OWL)^23^ file. This includes every one of the data fields (a.k.a. column headers) it encounters in the imported database.

**Figure 1.**
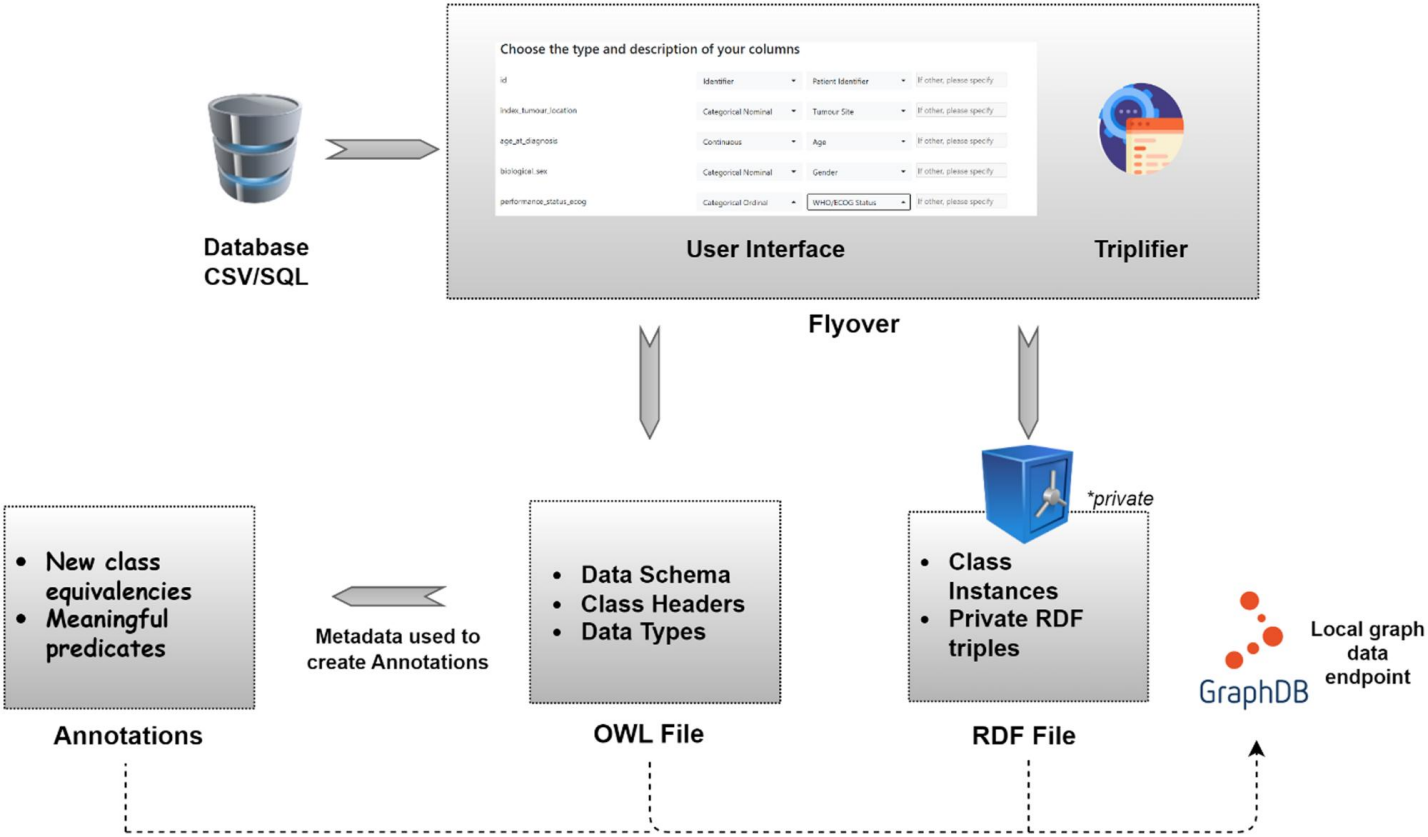
Illustration of the conversion workflow into FAIR data. The flyover package first processes the CSV or SQL data into RDF triples and an OWL schema. The GUI then prompts the data owner to add some preannotations, for example, commonly used terminologies from a pull-down menu. The preannotations are written into the OWL file and saved into a GraphDB repository together with the RDF data. From the OWL file, classes and predicates from a public open ontology are added as local annotations, which are then also saved in the GraphDB repository. GUI = graphical user interface; FAIR = Findable-Accessible-Interoperable-Reusable; OWL = Web Ontology Language; RDF = Resource Descriptor Format.

The GUI then iterates through every data field that was extracted into the OWL schema. For each field, it will prompt the user to add initial metadata, such as data type (continuous, discrete, categorical ordinal, or patient identifier). The user may attach preselected definitions to their data headers (eg, biological sex, tumour staging) and categorical codings where it matches, or else provides additional supplementary explanation in an open text box. These user inputs are directly appended to the OWL file. Both TTL and OWL files are automatically saved as a graph database application called GraphDB. This data conversion step is explained visually in Figure 1. Example fragments of output from *triplifier* are given in Figure S1.

The RDF contains the processed patient data, so this must always be kept private. However, the OWL file only contains the schema of the database, such as the column headers, and the user inputs through the aforementioned GUI. Since this is not privacy-sensitive, the local investigator can physically share only the OWL file with an external researcher and/or a remote principal investigator, for example, through any secure file service of their choice. The OWL file was then used to construct a linking of semantic meaning, from each local investigator’s data fields and native data coding, to a terminology applied throughout the entire FL consortium.

**Figure 2.**
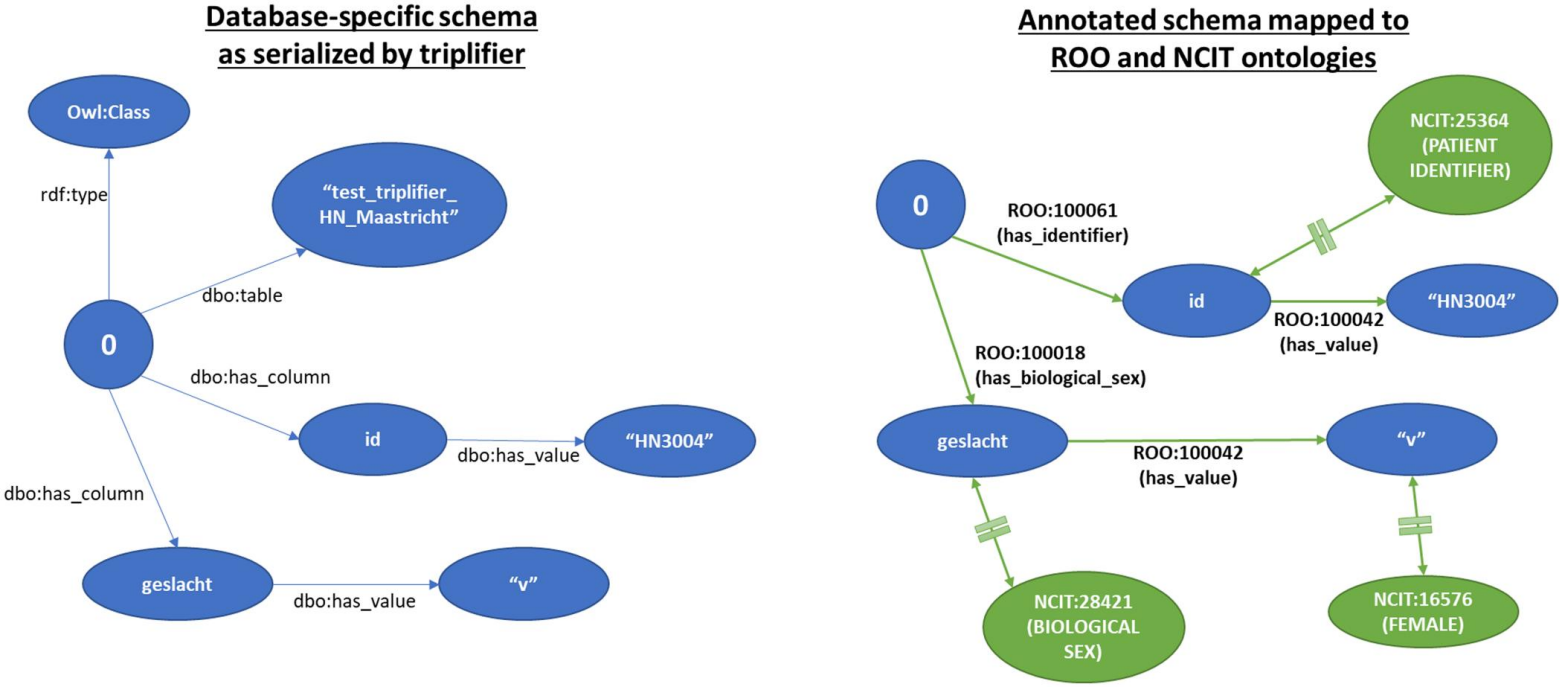
Examples showing knowledge graph of a patient “0” with class ID and biological sex. The image on the left is from triplifier after the imported data has been converted to RDF triple format. On the right, is the image of the knowledge graph for the same patient after the annotation graph has been added. Double-sided green arrows with double-crossing bars are the predicate owl: equivalentClass. RDF = Resource Descriptor Format.

In this working proof, we made a semantic harmonization of meaning in the data, across 5 different datasets with entirely different schema, using publicly accessible ontologies/terminologies—the Radiation Oncology Ontology (ROO)^24^ and the National Cancer Institute Thesaurus (NCIt).^25^ This is illustrated in Figure 2, where the original data in RDF (blue bubbles) and the original relationships (blue arrows) have been supplemented by semantic class equivalencies from the NCIt (green bubbles) and relationships (known as predicates) from the ROO (green arrows). Files that contain the semantic mappings (we named them “annotation.local”) are then returned by the principal investigator (PI)/lead researcher to each of the local investigators to keep within their respective data stations, 1 unique annotation.local file per data station. The annotation.local file is stored as a graph object in the local GraphDB instance.

We also had the radiotherapy CT and RTSTRUCT data, from which we extracted 103 radiomics features per subject from each patient’s primary GTV. Software for the image-based features was as described elsewhere by Shi et al^26^ and the PyRadiomics^27^ software packages. We converted the radiomics to RDF and created a *radiomics_annotation.local* linking file, same as described above, using the Radiomics Ontology.^28^ For this working proof, we only hand-picked 5 radiomics features of interest identified in previous studies of prognostic performance and vulnerabilities^29^^,^^30^; there was no radiomics feature selection or elimination study performed within this present work.

### Creating an FL collaboration

**Figure 3.**
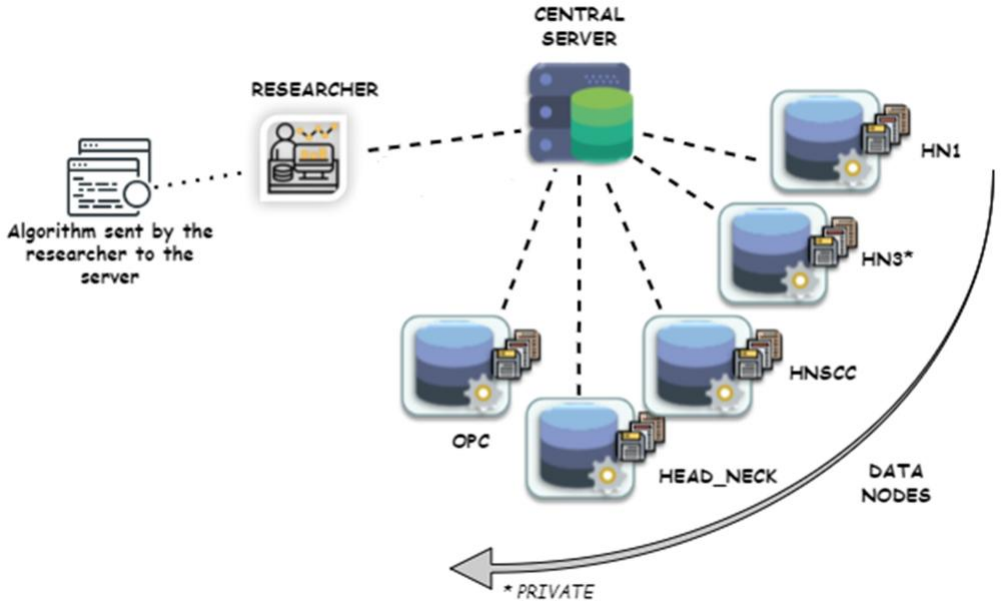
Schematic illustration of the Vantage6 infrastructure used in this work to show a likely clinical use case. Datasets OPC, HEAD_NECK, HNSCC, and HN1 are open access, but HN3 dataset is private at the time of this work.

We implemented Vantage6 as an open-source infrastructure that conforms to the PHT manifesto.^31^^,^^32^ A PHT network instantiates a hub-and-spoke topology (see schematic in Figure 3) such that direct peer-to-peer connections are prohibited. The only allowed communications run between data stations and a trusted central aggregation server. A researcher with valid account credentials is allowed to log into this server and request that the data stations execute an algorithm. The aforementioned data stations, upon receiving the request message, automatically and individually pull identical copies of an executable algorithm from a specified repository. We use Docker^®^ containers to deploy algorithms,^33^ and task orchestration between server and data stations is fully automated by Vantage6.

### Federated data exploration

Once data has been processed FAIR by local investigators and the data stations connected as abovementioned, we deployed a simple a federated dashboard to show how a member of the collaboration will be able to explore case mix between datasets (eg, via variable such as age, sex, and tumour staging) using python PlotLy and Dash libraries. The dashboard consists of a user interface that allows the user to select datasets and case mix variables. As the user interacts with the interface, we permit federated queries to be updated in real time through the Vantage6 connectivity between data stations, and the graphical display updates automatically when new results are returned. In accordance with PHT principles, no individual patient data are shared, the dashboard only sends requests for partial cohort summaries from each data station and pools the descriptive statistics together. The federated query uses the semantic linking in each data station’s *annotations.local* file, which allows a common query to be executed in 5 divergent datasets.

### Federated training of Cox models using clinical and radiomic features

After exploring the clinical case mix in the aforementioned dashboard, we selected a subset of primary OPC nonmetastatic cases treated with chemo-radiotherapy to train a federated Cox Proportional Hazards (CPH) regression^6^ for overall survival (OS). We attempted to reduce to parsimonious multivariable models via step-wise backward regression.

First, we trained using the clinical case mix variables only (hereafter our “C-Model”) to predict OS. We separately used radiomic features only to fit a federated CPH model for OS (hereafter “R-Model”). For radiomics, we built in a Box-Cox transformation, centring and *z*-scaling into the federated CPH model. To combine the C-Model and R-Model, we used Vantage6 to request each data station to compute linear predictors using the prespecified coefficients of the C-Model and R-Model. These were not transmitted and only stored locally in the data station. We then repeated the federated training of a CPH prognostic model with only linear predictors of the C-Model and R-Model as inputs, hereafter we call this the (“CR-Model”).

To assess the discrimination performance of each of the C-Model, R-Model, and CR-Model, we also distributed a validation algorithm to compute the dataset-by-dataset Harrell Concordance Index (HCI). We were also able to apply the “leave-one-dataset-out” iterative procedure of internal-external validation as recommended by Steyerberg and Harrell.^34^

## Results

One of the FAIR principles is to assign globally unique and persistent locators (eg, web URLs) to both metadata and (if available) the actual data (ie, principle F1). The public datasets we have used are unambiguously referenced using a persistent Digital Object Identifier (DOI). The CT and RTSTRUCT for these public datasets can be obtained online. Though private dataset HN3 is not openly accessible, we can openly share a readable description of the dataset, the OWL schema descriptor file, and its semantic ontology annotations (“annotation.local”) in an open Zenodo data catalogue with a unique persistent DOI for this metadata. This DOI is cross-referenced with this open-access article and with our open-access software repository. Along with the online materials accompanying this work, we have provided python scripts that generate *annotation.local* files for the public head and neck cancer datasets, which can be openly reused.

**Figure 4.**
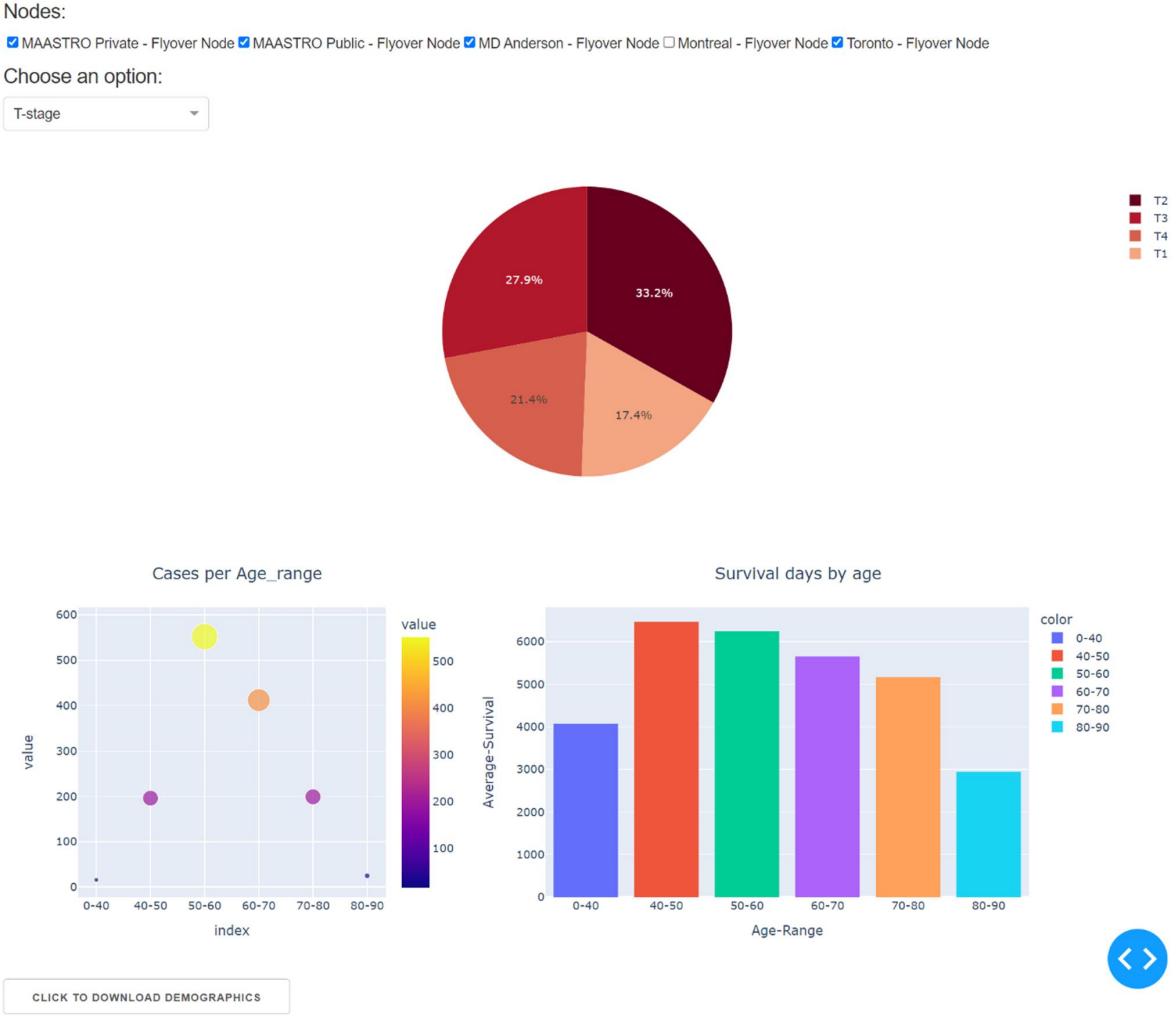
Screen capture of a simple prototype dashboard. Cohort summaries from each of the 5 geographically dispersed FAIR data repositories were retrieved via SPARQL query in the Vantage6 infrastructure. The dashboard allows a user to choose (via checkboxes along the top of the page) which of the cohorts to aggregate in the summary statistics, along with a clinical attribute to display (here tumour staging is shown as example). The dashboard user may also download the summary statistics (as a CSV file) from the Vantage6 server using the button at the bottom left corner. FAIR = Findable-Accessible-Interoperable-Reusable; SPARQL = Semantic Web Query and Retrieval Language.

A snapshot of the interactive visual dashboard is given in Figure 4. This allows a dashboard user to see in 1 glance what type of patients are available across the dispersed FAIR data stations. It is also possible to download a case-mix aggregated summary directly from the dashboard as a CSV file. The latter can be easily reformatted and is included here in Table 1.

**Table 1.** Clinical case-mix summary statistics from each of the 5 data nodes.

	HN1	HNSCC	OPC	HEAD-NECK	HN3
**Sample size**	137	492	606	298	165
**Age in years**					
Mean	61.9	57.8	60.5	63.3	62.6
Range	44-83	28-87	33-89	18-90	29-84
**Sex**					
Female	26	69	125	71	43
Male	111	423	481	227	122
**Tumour stage**					
T1	35	92	103	39	14
T2	32	203	198	109	31
T3	24	117	183	94	68
T4	46	80	122	46	52
Tx	–	–	–	10	–
**Nodal stage**					
N0	60	45	101	59	48
N1	16	53	61	40	45
N2	58	378	397	180	54
N3	3	16	47	19	18
Nx	–	–	–	–	–
**Metastasis stage**					
M0	136	492	606	294	165
M1	1	0	–	0	0
Mx	–	–	–	4	–
**Overall stage (7th ed.)**					
I	24	3	11	4	–
II	11	16	38	27	–
III	23	67	85	61	–
IV	79	406	472	204	–
Unspecified	–	–	–	2	–
**Tumour location**					
Nasopharynx	–	–	–	28	–
Oropharynx	88	492	606	203	63
Hypopharynx	–	–	–	13	31
Larynx	49	–	–	45	64
Unknown	–	–	–	9	–
**HPV status**					
Positive	23	248	356	78	34
Negative	58	44	143	46	29
Unknown	56	200	107	174	102
**Radiotherapy type**					
Radiotherapy	100	57	309	48	104
Chemoradiotherapy	37	435	297	250	61
**Survival status**					
Censored	63	376	347	242	77
Deceased	74	116	259	56	88

The C-Model was developed on a subset of 1492 subjects treated by (chemo)-radiotherapy for primary OPC (HN1 = 88, HNSCC = 492, OPC = 606, HEAD-NECK = 203, and HN3 = 63). After matching primary gross tumour volume (GTV) for these subjects and linking to their previously computed radiomic features, the R-Model was constructed from 1321 subjects (HN1 = 80, HNSCC = 396, OPC = 582, HEAD-NECK = 203, and HN3 = 60). The difference in sample size was due to either not having some patient’s preprocessed radiomics features beforehand, or there was no primary GTV delineated near the oropharynx.

Global coefficients of the fully trained C-Model and R-Model can be retrieved from the central server and are presented in Figures S2 and S3, respectively. The infrastructure was flexible enough to support the local computation of linear predictors using prespecified coefficients of the C-Model and R-Model, and then, we were able to train a combined hybrid clinical-radiomics (CR-Model) using the linear predictors. The results of the CR-Model are given in Figure S4.

HCI results from internal validation and cyclical internal-external validation (fitted with 4 *other* nodes, validated in the excluded node, as an estimate of potentially over-optimistic discrimination) are summarized in Table S1. The federated CPH performance varied from 1 dataset to another, but there was no suggestion of over-optimism. Overall, the C-Model (HCI range 0.62-0.73) performed better than the R-Model (HCI range 0.61-0.68), and the hybrid CR-Model (HCI range 0.60-0.72) was not improved relative to the C-Model.

## Discussion

In this work, we have developed tools that will significantly help clinicians and researchers—who wish to collaborate with each other in the PHT FL paradigm—to make their data FAIR. This data preparation step needs to be done fully on-site by a local investigator, who has ultimate control of the data, understands their own database, and is able to help a remote researcher to create semantic interoperability with their dataset. Once the FAIR data stations are prepared, an infrastructure (ie, the trains and tracks) such as Vantage6 can be instantiated that permits FL between a remote researcher and the local investigators. We showed that data exploration and graphical visualization of clinical case mix through a federated dashboard are possible, without the need to transmit individual patient-level data outside of the data stations. We used a well-established privacy-preserving algorithm to show how to construct a methodologically robust prognostic modelling study within the PHT paradigm incorporating training, internal validation, and internal-external loop validation.

It is important to observe that our general approach to data is generalizable to other clinical learning tasks, such as for machine learning or training of deep learning neural network, because the entire infrastructure is “model agnostic.” That is, as long as the data is prepared FAIR to the extent that a given executable algorithm can find what it needs, then our federated CPH can be replaced with some other algorithm. The necessary job is to package some desired algorithm as a Docker^®^ container and then post it within a PHT collaboration network as we have done here. Likewise, our approach for making data FAIR is also “disease agnostic”—the steps involved in making any other kind of disease data FAIR will be the same as we have described.

Although not explicitly illustrated in Figure 3 for brevity, directed acyclic graphs of arbitrarily complex relationships in the data could be constructed out of RDF, for example, each subject may have 1 or more neoplasms, and each neoplasm is associated with its own set of diagnostic findings, cancer staging, follow-ups, etc., that is disjoint from other previous, concurrent, or subsequent neoplasms. Where needed, numerical values may be supplemented by extra predicates and classes indicating the exact units of the measure, for example, age in years, and follow-up time as intervals of days, or months, or years.

Specific interpretations of some lesser-known variables, such as nicotine exposure, can be encoded as a class with smoking cigarette and chewing tobacco as lower-level subclasses of nicotine exposure, where required. This can also be accompanied by a subclass for quantity used, such as number of pack-years of cigarettes. Similarly, one may define a superclass “T4” for tumour staging, with T4a and T4b as subclasses. In this case, searching for T4 will return all subjects with T4, T4a, and T4b, whereas querying for T4a returns only those subject with T-stage corresponding only to 4a. Examples of knowledge embedding of clinical hierarchical relationships into the data graph have been included for the reader in the online materials.

For enhancing reusability of local databases, our approach of converting data into schema-independent RDF objects and then adding semantic linking using a study-specific *annotations.local* can be repeated an unlimited number of times, but the underlying OWL and RDF objects will never change. For instance, instead of the ROO or NCIT, we could add another annotations layer linking to the hierarchies and terminologies in the OHDSI (*Observational Health Data Sciences and Informatics*)^7^ common data model. Importantly, both annotations will co-exist side by side on top of the same data; it is only the federated query that must specify which annotation has the semantic linkage that is referenced by a specific algorithm or model.

At present, we did not impose constraints on what kinds of classes can be joined by a given predicate, but this will be very useful to implement in future work. Specifically, the Shapes Constraint Language (SHACL) can be used,^35^ to insert structural constraints on the kinds of data graphs that can be assembled as well as define requirements for data quality audits. A related approach based on OWL, RDF, and data quality assurance based on SHACL has been implemented by the Swiss Personalized Health Network^36^ public health data. An example of such a SPARQL query is provided in the Supplementary section.

For future work, we propose that privacy-preserving methods to check for data quality and potential confounders are significant capabilities that ought to be added to federated data exploration dashboards. In the results of the C-Model (Figure S2), chemoradiotherapy is strongly associated with a good prognosis, but this does not account for elderly patients and those in poor overall health not being offered chemoradiotherapy as a treatment option. This is a clinical confounder that may be detected if such data were uniformly collected everywhere and visualization of intervariable correlations were possible via a dashboard. Likewise, the R-Model performance might be improved if we had redone feature selection and feature elimination, but we have not yet included those tools in the present work. Those objectives were not the principal messages of this study. The C-Model, R-Model, and CR-Model illustrate our working principles but were not intended to be clinically usable prognostic models at the present time.

The federated CPH model^6^ does not exchange any patient characteristics that might reasonably lead to reidentification, but it does compile a list of unique event times occurring in all training datasets. This is generally not thought of as directly reidentifiable, because the same information would be easily extractable from any publicized Kaplan-Meier curve from any clinical study; therefore the list of event times compiled by the federated CPH algorithm is not any more “identifiable” than publishable results in the public domain. However, if there are only 2 data stations in the collaboration and one of them contains publicly known data with event times, it may be possible to deduce some of the unique event times in the unpublished dataset. However, to actually reidentify a subject from only an event time requires gaining access to additional data.

However, in very small datasets and extremely rare events such as certain types of childhood cancers, the chance of locating other kinds of data (such as social media posts) to match with an event time might be increased. In mitigation of such circumstances, future work in FL design might be enhanced with outlier data detection and additional safeguards against repetitive-inductive dashboard queries that try to isolate the summary statistics of single individuals.

FL experiments on real-world cancer data have only been reported a handful of times,^1–4^ but the domain is maturing steadily. Sullivan et al^37^ predicted that it would be the combination of ethical/legal issues, political barriers, and administrative limitations—but not especially technical know-how—that would limit data sharing. Hallock et al^38^ offered “federated (data) networks as a solution to enable access to diverse data sets and tackle known and emerging health problems.” These authors state that uneven application of privacy legislation and divergent legal interpretation have paradoxically led to data becoming even more siloed within institutional and national boundaries, and FL could help bridge certain siloes. The World Economic Forum published a guidance document outlining 8 steps towards sharing sensitive health data in a federated data consortium model.^39^ This report specifically refers to the need for establishing and sustaining trust in a federated data consortium, jointly determining the problems to be solved with a federated approach, creating an organizational and governance model that addresses resourcing, leadership, funding, and policy gaps, and—of particular interest to the aim of this paper—deploying tools that can structure and prepare the data accordingly.

To date, most research in FL to date consisted of sandbox simulations or focused heavily on the model development step, and only a handful of FL experiments have actually been executed in the real world on real cancer patients’ data.^1–4^^,^^40^ These studies give paltry insight into their data preparation tools, and we infer from the published text that all datasets have to be entered into the same schema, with exactly the same header nomenclature and recoded to the same dictionary. This addresses the short-term need for interoperability but risks losing finer context from the data and does not support long-term reusability of the same data on new clinical questions. In contrast, as our above hypothetical about T-stage 4, 4a, and 4b illustrates, our way of managing the data can flexibly adapt to different analyses without needing to change the underlying original data.

Consortium governance frameworks and legal templates have recently been made publicly accessible,^11^ and such frameworks are rapidly maturing through use in major EU Horizon-funded projects such as STRONG-AYA,^41^ EUCanImage,^42^ and CCI4EU.^43^ Some of the ongoing challenges to implementation of FL include building trust between would-be consortium partners, lack of political incentives to share data, coming to terms with use/reuse of highly heterogenous data and better tools for assessing data quality during the exploratory phase.

## Conclusions

FL is a potentially powerful tool for uncovering latent value in a vast amount of oncology data available worldwide. Experimental studies in FL focus heavily on the algorithms and training of models, but few details are given about data preparation. While governance frameworks and legal templates are now becoming more mature in terms of facilitating real-world federated data networks, more attention is needed to prepare data so as to maximize the contextual richness of institutional data, and at the same time make the data amenable for multiple different investigations. We provide here a set of public open-access software tools aiming to help institutional clinicians and global researchers make data FAIR and thus ready for use in FL-based data-sharing consortia. Tools to explore data quality and to detect potential bias/confounding would be significant capabilities that should be added to federated data exploration dashboards in the future.

## Supplementary Material

ubae005_Supplementary_Data
