# Supplementary material for: Making head and neck cancer clinical data Findable-Accessible-Interoperable-Reusable to support multi-institutional collaboration and federated learning
Source: BJR Artif Intell. 2024 Mar 6;1(1):ubae005. doi: 10.1093/bjrai/ubae005 (PMC13045714; doi:10.1093/bjrai/ubae005)
Supplement: ubae005_Supplementary_Data [file ubae005_supplementary_data.docx]

SUPPLEMENTARY MATERIALS

**Software availability and requirements**

Project name: Flyover (tagged release: v1.0, preprint demonstration project branch: MedRxiv)

Project home page: <https://github.com/MaastrichtU-CDS/projects_flyover_project>

Zenodo Repository DOI: <https://doi.org/10.5281/zenodo.7190551>

Operating system(s): Platform independent

Recommended processor, memory and storage: Intel i5 – 8^th^ gen equivalent or higher, 8GB RAM, at least 16GB storage.

Programming language: Python

Other requirements: Either Docker Desktop (Windows/MacOS) or Docker Engine (Linux), plus Docker Compose 3.7.

License: Docker Desktop/Engine is licensed as part of either a free or paid Docker subscription (<https://www.docker.com/pricing/faq/>). Jupyter/base-notebook image is distributed under a Modified BSD License. GraphDB Free Edition is distributed as freeware.

Restrictions on use by non-academics: Creative Commons By Attribution Non Commercial only version 4.0 International (CC BY-NC 4.0).

**List of abbreviations**

CPH: Cox Proportional Hazards

CSV: Comma-Separated Values

CT: Computed (x-ray) tomography

DOI: Digital Object Identifier System

EU: European Union

FAIR: Findable Accessible Interoperable Reusable

GDPR: General Data Protection Regulation

GTV: Gross Tumor Volume

HTTPS: Hypertext Transfer Protocol - Secure

IRB: Institutional review board

NCIt: National Cancer Institute Thesaurus

O-RAW: Ontology-guided Radiomics Analysis Workflow

OHDSI: Observational Health Data Sciences and Informatics

OPC: Oropharyngeal Cancer

OWL: Web Ontology Language

PET: Positron emission tomography

PHT: Personal Health Train

ROO: Radiation Oncology Ontology

SHACL: Shapes Constraint Language

SPARQL: SPARQL Protocol and RDF Query Language

SQL: Structured Query Language

TCIA: The Cancer Imaging Archive

TTL: Terse RDF Triple Language

URI: Uniform Resource Identifier

**Declarations**

*Ethics approval and consent to participate*

Approval to use the private dataset HN3 for this investigation was granted by the Institutional Review Board (IRB) of MAASTRO Clinic, The Netherlands (reference P0415) as the TRAIN retrospective observational study protocol (ClinicalTrials.gov Identifier NCT04655469).

*Consent for publication*

Not applicable.

*Availability of data and materials*

Four public datasets were obtained from The Cancer Imaging Archive (TCIA) [30]. **RADIOMICS-HN1** [31] comprises clinical data, volumetric CT and PET of 137 patients with laryngeal carcinoma and OPC treated by RT alone or currently with either cisplatin or cetuximab and had been used as model validation dataset by Aerts et al. [32]. **HNSCC** contains clinical data and contrast-enhanced CT scans of 627 oropharyngeal cancer (OPC) patients [33]. **OPC-Radiomics** has clinical data and CT scans of 606 OPC subjects, treated by either radiotherapy or chemo-radiotherapy between 2005 and 2010 [34], that was part of a radiomics prognostication study [35]. **HEAD-NECK-PET-CT** [36] comprised 298 subjects with multiple subsites of HNC each with clinical descriptors, PET and planning CT, treated between April 2006 and November 2014, and was also the subject of a radiomics prognostication study [37]. The **HN3** dataset is not publicly available at the present time due to material that is potentially identifiable of an individual. Redacted patient data, subject to a data transfer agreement, is available from authors LW and FH upon reasonable request.

Ontologies used openly accessible in Bioportal, (<https://bioportal.bioontology.org/ontologies/ROO>, <https://bioportal.bioontology.org/ontologies/NCIT>, https://bioportal.bioontology.org/ontologies/RO).

*Authors' contributions*

VG performed all of the data processing, Python coding and statistical experimentation. VG prepared the majority of the manuscript text with assistance from JvS and LW. AC was mainly responsible for setting up the Vantage6 infrastructure, and assisted VG with the statistical experimentation. FH and FW are clinicians responsible for the data collections HN1 and HN3, and assisted with clinical interpretation of the statistical experiments. MW and SK were responsible for preparation of the OPSCC dataset and setup of the infrastructure in Toronto, and both assisted with the statistical experimentations. JK is a clinician who contributed to the visualization dashboard concept. AD and BH-K contributed significantly to editing of the manuscript. JvS and LW were jointly responsible for the overall supervision of the work, and for editing of the manuscript. All authors have inspected the manuscript prior to submission and given their consent to publication.

**Additional results and figures**


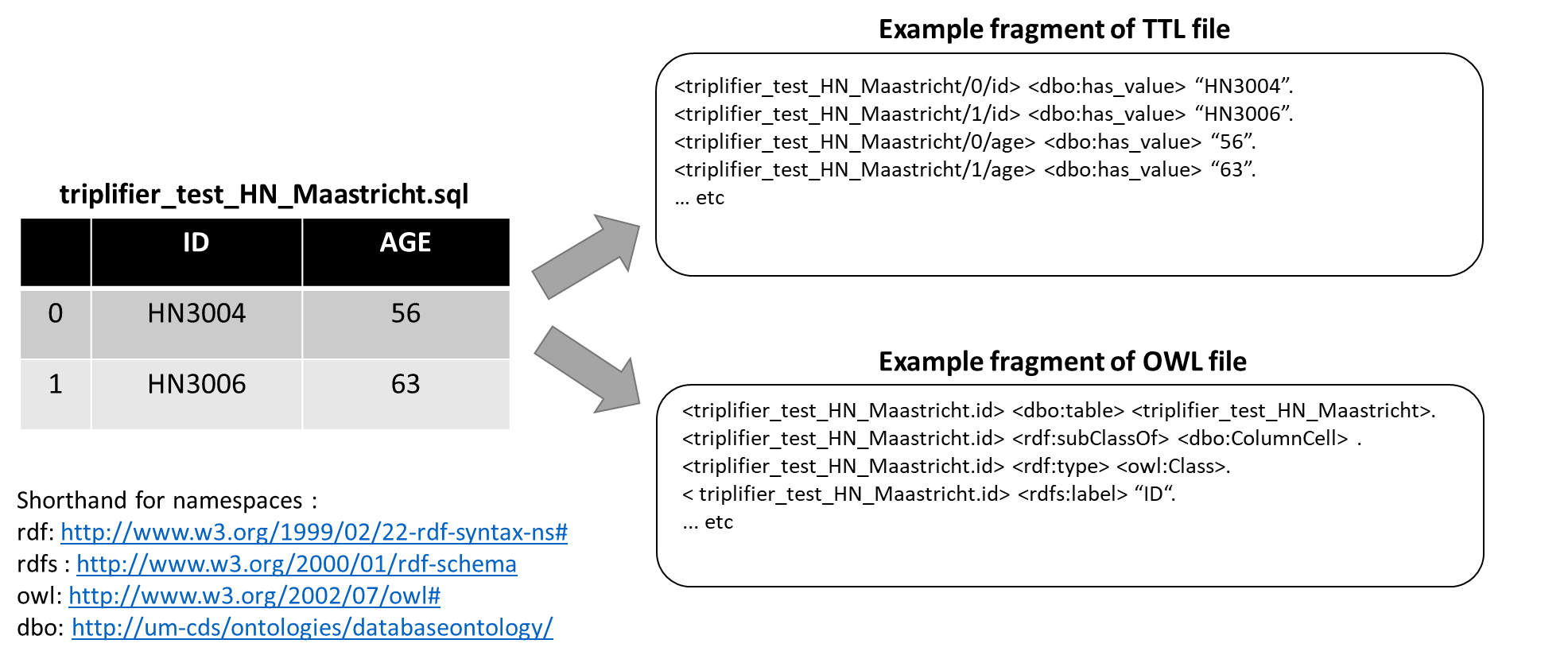


*Fig S1. Example showing the expected output of triplifier processing. A hypothetical input table is shown on the left. Namespace aliases (bottom left) are used to simplify and improve readability of the figure. A fragment of the serialized database contents is shown top right in the RDF file, and a part of the database schema is shown with a database-specific ontology in the OWL file at bottom right.*

**
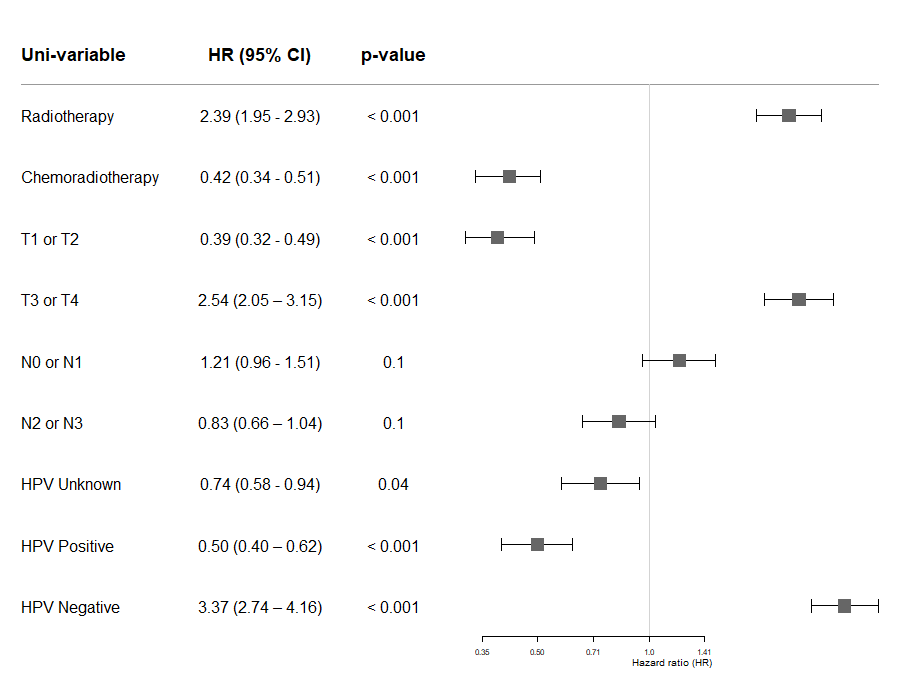
**

**
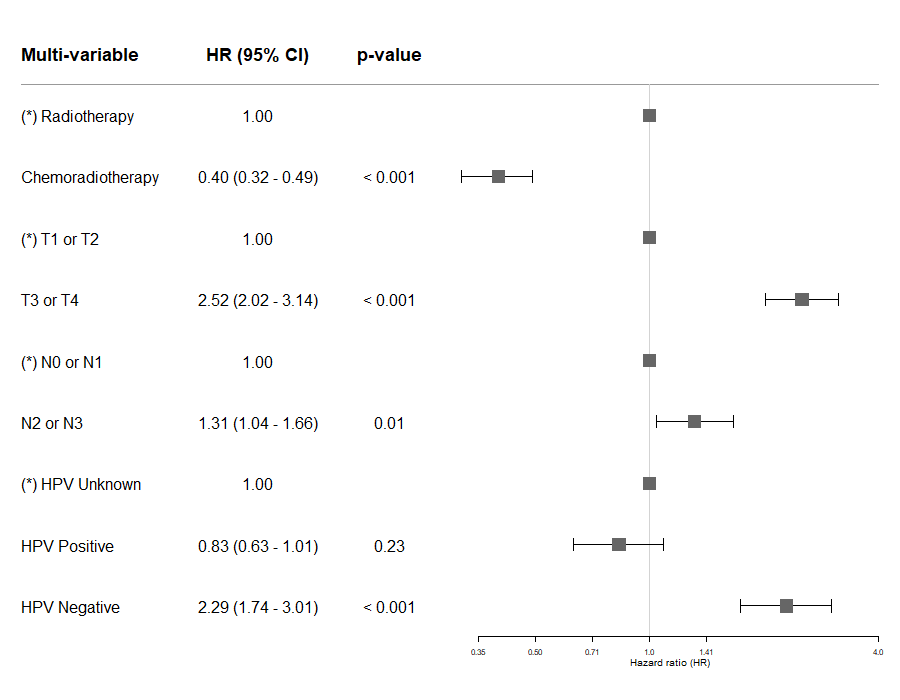
**

*Fig S2. Uni-variable (upper inset) and multi-variable Federated Cox regression with Clinical predictors only. Results are fitted over all 5 HNC datasets, indicating hazard ratio (HR) and 95% confidence intervals of the HR. First line treatment type, T stage and N stage were independently predictive of OS in the final C-Model. Human papilloma virus (HPV) tests were not consistently done for all OPC patients, hence there was a large proportion of “unknown” HPV status which obscured the prognostic importance of HPV positive versus HPV negative. For categorical predictors, the prefix (*) indicated which category was assumed as the reference value for the predictor.*


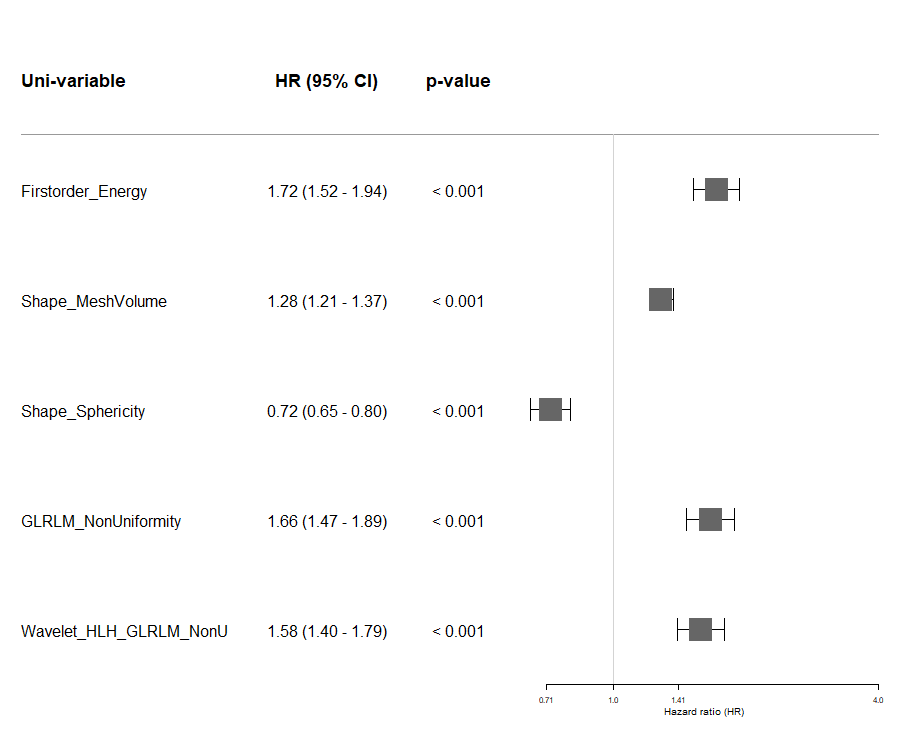


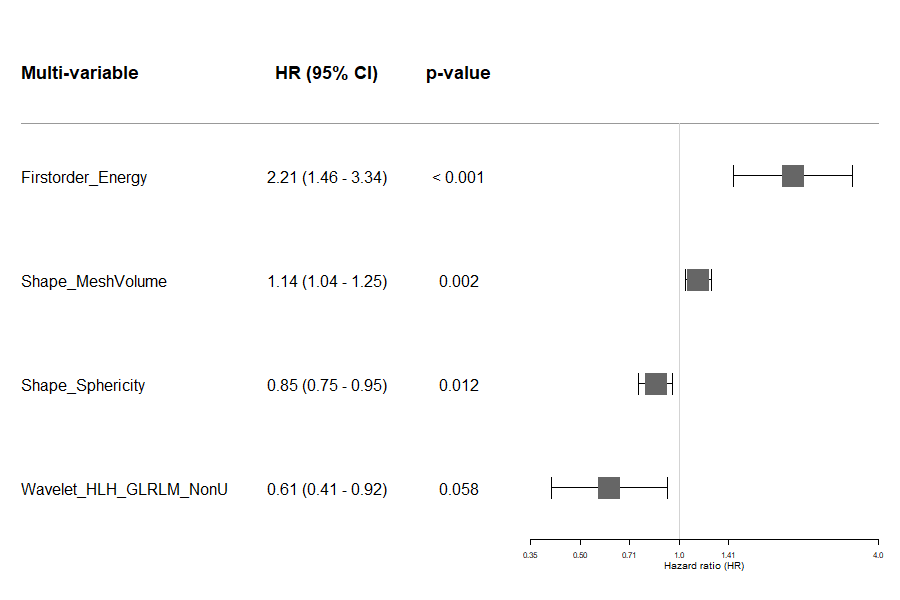


*Fig S3. Uni-variable (upper inset) and multi-variable Federated Cox regression with Radiomics predictors only. Results are fitted over all 5 HNC datasets, indicating hazard ratio (HR) and 95% confidence intervals of the HR. In the multi-variable fit, radiomics feature “GLRLM_NonUniformity” was clearly not an independent predictor and was thus dropped from the final multi-variable model. All radiomics features were continuous numerical variables.*


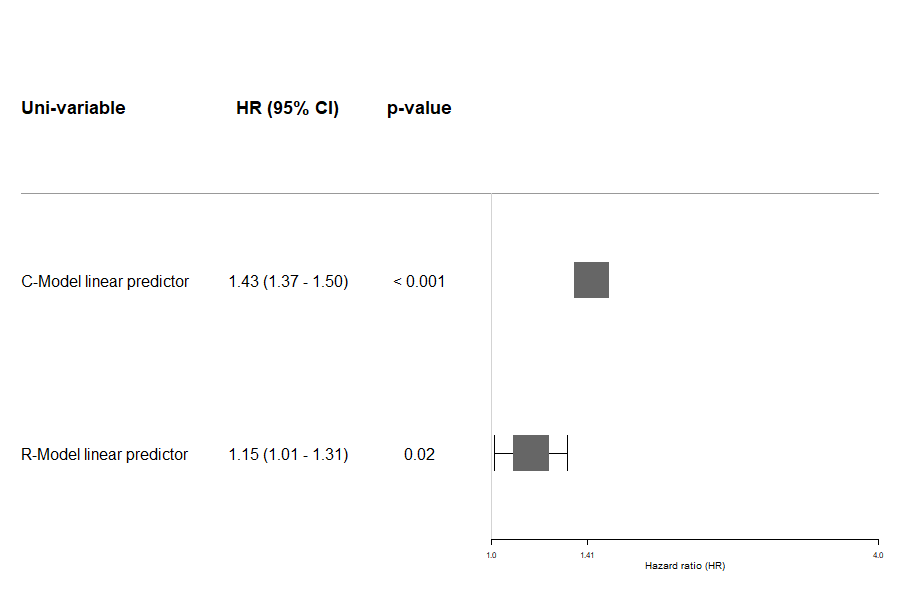


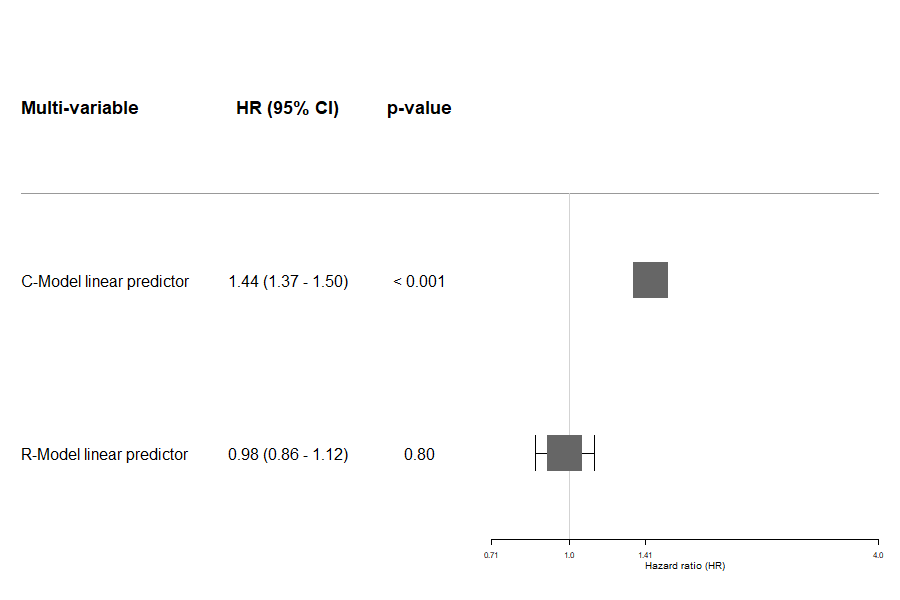


*Fig S4. Uni-variable (upper inset) and multi-variable Federated Cox regression using the linear predictors (LPs) from the C-Model and R-Model previously obtained. Results are fitted over all 5 HNC datasets, indicating hazard ratio (HR) and 95% confidence intervals of the HR. While the LPs of the individual models do support survival stratification by themselves, in combination it is found that the R-Model linear predictors are not an independent predictor for survival and do not have added value for prediction in the CR-Model.*

| *Dataset* | HCI : C-Model | HCI : R-Model | HCI : CR-Model |
| --- | --- | --- | --- |
| HN1 | **0.73** (0.72) | **0.63** (0.63) | **0.72** (0.72) |
| HNSCC | **0.63** (0.63) | **0.68** (0.68) | **0.64** (0.64) |
| HN-PET-CT | **0.62** (0.62) | **0.61** (0.60) | **0.60** (0.59) |
| OPC | **0.72** (0.72) | **0.65** (0.65) | **0.73** (0.73) |
| HN3 | **0.66** (0.66) | **0.65** (0.65) | **0.66** (0.66) |

*Table S1. Harrell concordance indices (HCIs) computed on each distributed dataset using the multivariable models C, R and CR. In bold, the model was fitted on all datasets and then tested in the one dataset indicated in the leftmost column. To estimate magnitude of over-optimistic model performance, we fitted the coefficients for the selected features in 4 other datasets, then computed the HCI in the one dataset left out. The number in parentheses represents the HCI where the one dataset left out is indicated in the leftmost column. Therefore the divergence between the value in bold and the value in the adjacent parentheses is an estimate of the over-optimism.*

**Additional files**

*Example SPARQL query*

The following query is built to retrieve patient’s ID and age from an annotated RDF database in the following manner. The query first looks for the instances of classes that include the predicates P100061 and P100000 (which resolves to has_identifier and has_age from ROO respectively), which is added on top of the common dbo predicate “has_column” (as seen in the OWL) in the annotation graph. Following that, it finds the cells for those instances by matching them to the dbo predicate has_cell and finally the values in those cells are fetched by using the dbo predicate has_value. The namespace “dbo” is in the structure of the output TTL file and can be viewed using the OWL schema.

PREFIX dbo: <http://um-cds/ontologies/databaseontology/>

PREFIX roo: <http://www.cancerdata.org/roo/>

SELECT ?patientID ?agevalue

WHERE

{

?tablerow roo:P100061 ?ID. #returns instances with predicates roo:P100061 (has_identifier)

?ID dbo:has_cell ?IDcell. #returns the triples with cells for those instances

?IDcell roo:P100042 ?patientID. #gets the values in those cells, which gives the patient IDs

?tablerow roo:P100000 ?age. #returns instances with predicates roo:P100000 (has_age)

?age dbo:has_cell ?agecell. #returns the triples with cells for those instances

?agecell roo:P100042 ?agevalue. #gets the values in those cells, which gives the age values

}
